# Association of glomerular hyperfiltration with mortality in stroke: an analysis using pooled individual patient data

**DOI:** 10.1093/esj/aakag042

**Published:** 2026-05-11

**Authors:** Philip S Nash, Gareth Ambler, Jonathan G Best, Duncan Wilson, Houwei Du, Rustam Al-Shahi Salman, Hans Rolf Jäger, Gregory Y H Lip, Simon Fandler-Höfler, Sebastian Eppinger, Kaori Miwa, Masayuki Shiozawa, Masatoshi Koga, Martina B Goeldlin, Morin Beyeler, Philipp Bücke, Marwan El-Koussy, Heinrich P Mattle, Leonidas D Panos, Dianne H K van Dam-Nolen, Florian Dubost, Jeroen Hendrikse, M Eline Kooi, Werner Mess, Paul J Nederkoorn, Nicolas Christ, Maximilian Bellut, Sarah Gunkel, Chris Karayiannis, John Van Ly, Shaloo Singhal, Lee-Anne Slater, Young Dae Kim, Keon-Joo Lee, Jae-Sung Lim, Hideo Hara, Masashi Nishihara, Jun Tanaka, Masaaki Yoshikawa, Derya S Demirelli, Zeynep Tanriverdi, Natan M Bornstein, Jeremy Molad, Einor Ben Assayag, Hen Hallevi, Ender Uysal, Shelagh B Coutts, Francesca Chappell, Stephen Makin, Henry Mak, Kay-Cheong Teo, Debbie Y K Wong, Lisa Hert, Marta Kubacka, Philippe Lyrer, Alexandros Polymeris, Benjamin Wagner, Annaelle Zietz, Jill Abrigo, Cyrus Cheng, Winnie Chu, Thomas W H Leung, David Seiffge, Urs Fischer, Simon Jung, Christian Enzinger, Thomas Gattringer, Daniel Bos, Kazunori Toyoda, Felix Fluri, Thanh Phan, Velandai Srikanth, Ji Hoe Heo, Hee-Joon Bae, Yusuke Yakushiji, Dilek N Orken, Eric E Smith, Joanna M Wardlaw, Gary K K Lau, Stefan T Engelter, Nils Peters, Yannie Soo, Rob J Simister, David C Wheeler, David J Werring, Tae-Jin Song

**Affiliations:** UCL Stroke Research Centre, Department of Translational Neuroscience and Stroke, University College London Queen Square Institute of Neurology, London, United Kingdom; Comprehensive Stroke Service, National Hospital for Neurology and Neurosurgery, University College London Hospitals NHS Trust, London, United Kingdom; Department of Statistical Science, University College London, London, United Kingdom; UCL Stroke Research Centre, Department of Translational Neuroscience and Stroke, University College London Queen Square Institute of Neurology, London, United Kingdom; UCL Stroke Research Centre, Department of Translational Neuroscience and Stroke, University College London Queen Square Institute of Neurology, London, United Kingdom; UCL Stroke Research Centre, Department of Translational Neuroscience and Stroke, University College London Queen Square Institute of Neurology, London, United Kingdom; Department of Neurology, Fujian Medical University Union Hospital, Fuzhou 350001, China; Institute for Neuroscience and Cardiovascular Research at The University of Edinburgh, Edinburgh, United Kingdom; Lysholm Department of Neuroradiology and the Neuroradiological Academic Unit, Department of Translational Neuroscience and Stroke, UCL Queen Square Institute of Neurology, Queen Square, London, United Kingdom; Liverpool Centre for Cardiovascular Science at the University of Liverpool, Liverpool John Moores University and Liverpool Heart & Chest Hospital, Liverpool, United Kingdom; Department of Clinical Medicine, Aalborg University, Aalborg, Denmark; Department of Cardiology, Lipidology and Internal Medicine with Intensive Coronary Care Unit, Medical University of Bialystok, Bialystok, Poland; Department of Neurology, Medical University of Graz, Graz, Austria; Department of Neurology, Medical University of Graz, Graz, Austria; Department of Cerebrovascular Medicine, National Cerebral and Cardiovascular Center, Suita, Osaka, Japan; Department of Cerebrovascular Medicine, National Cerebral and Cardiovascular Center, Suita, Osaka, Japan; Department of Cerebrovascular Medicine, National Cerebral and Cardiovascular Center, Suita, Osaka, Japan; Department of Neurology, University Hospital Inselspital Bern, University of Bern, Bern, Switzerland; Department of Neurology, University Hospital Inselspital Bern, University of Bern, Bern, Switzerland; Department of Neurology, University Hospital Inselspital Bern, University of Bern, Bern, Switzerland; Department of Diagnostic and Interventional Neuroradiology, University Hospital Inselspital Bern, University of Bern, Bern, Switzerland; Department of Neurology, University Hospital Inselspital Bern, University of Bern, Bern, Switzerland; Department of Neurology, University Hospital Inselspital Bern, University of Bern, Bern, Switzerland; Department of Radiology and Nuclear Medicine, Erasmus MC, Rotterdam, the Netherlands; Biomedical Imaging Group Rotterdam, Department of Radiology and Nuclear Medicine, Erasmus University Medical Centre, Rotterdam, the Netherlands; Department of Radiology, University Medical Centre Utrecht, Utrecht University, Utrecht, the Netherlands; Department of Radiology and Nuclear Medicine, CARIM Cardiovascular Research Institute Maastricht, Maastricht University Medical Center (MUMC+), Maastricht, the Netherlands; Department of Clinical Neurophysiology, Maastricht University Medical Centre, Maastricht, the Netherlands; Department of Neurology, Amsterdam University Medical Centres, Location AMC, Amsterdam, the Netherlands; Department of Neurology, University Hospital of Würzburg, Würzburg, Germany; Department of Neurology, University Hospital of Würzburg, Würzburg, Germany; Department of Neurology, University Hospital of Würzburg, Würzburg, Germany; Peninsula Clinical School, Peninsula Health, Monash University, Melbourne, Australia; Stroke and Ageing Research Group, School of Clinical Sciences at Monash Health, Monash University, Melbourne, Australia; Stroke and Ageing Research Group, School of Clinical Sciences at Monash Health, Monash University, Melbourne, Australia; Department of Interventional Radiology, Monash Health, Clayton, Victoria, Australia; Department of Neurology, Yonsei University College of Medicine, Seoul, South Korea; Department of Neurology, Seoul National University Bundang Hospital, Seoul National University College of Medicine, Seongnam, Republic of Korea; Department of Neurology, Asan Medical Center, University of Ulsan College of Medicine, Seoul, Republic of Korea; Division of Neurology, Department of Internal Medicine, Saga University Faculty of Medicine, Nabeshima, Saga, Japan; Department of Radiology, Saga University Faculty of Medicine, Nabeshima, Saga, Japan; Division of Neurology, Department of Internal Medicine, Saga University Faculty of Medicine, Nabeshima, Saga, Japan; Division of Neurology, Department of Internal Medicine, Saga University Faculty of Medicine, Nabeshima, Saga, Japan; Department of Neurology, Sisli Hamidiye Etfal Teaching and Research Hospital, University of Health Sciences, Istanbul, Turkey; Department of Neurology, İzmir Katip Çelebi University Atatürk Education and Research Hospital, İzmir, Turkey; Department of Neurology, Tel-Aviv Sourasky Medical Center, Tel-Aviv, Israel; Faculty of Medicine, Tel-Aviv University, Tel-Aviv, Israel; Department of Neurology, Tel-Aviv Sourasky Medical Center, Tel-Aviv, Israel; Faculty of Medicine, Tel-Aviv University, Tel-Aviv, Israel; Department of Neurology, Tel-Aviv Sourasky Medical Center, Tel-Aviv, Israel; Faculty of Medicine, Tel-Aviv University, Tel-Aviv, Israel; Sagol School of Neuroscience, Tel Aviv University, Tel Aviv, Israel; Department of Neurology, Tel-Aviv Sourasky Medical Center, Tel-Aviv, Israel; Faculty of Medicine, Tel-Aviv University, Tel-Aviv, Israel; Department of Radiology, Saglık Bilimleri University, Sisli Etfal Education and Research Hospital, Istanbul, Turkey; Calgary Stroke Program, Department of Clinical Neurosciences, Radiology and Community Health Sciences, Hotchkiss Brain Institute, University of Calgary, Calgary, Alberta, Canada; Centre for Clinical Brain Sciences, Edinburgh Imaging, and UK Dementia Institute at the University of Edinburgh, Edinburgh, United Kingdom; Institute of Applied Health Sciences, University of Aberdeen, Aberdeen, United Kingdom; Department of Diagnostic Radiology, The University of Hong Kong, Hong Kong, Hong Kong; Division of Neurology, Department of Medicine, The University of Hong Kong, Hong Kong, Hong Kong; Division of Neurology, Department of Medicine, The University of Hong Kong, Hong Kong, Hong Kong; Department of Neurology and Stroke Centre, University Hospital Basel and University of Basel, Basel, Switzerland; Stroke Center Klinik Hirslanden, Hospital Zürich, Zürich, Switzerland; Department of Neurology and Stroke Centre, University Hospital Basel and University of Basel, Basel, Switzerland; Department of Neurology and Stroke Centre, University Hospital Basel and University of Basel, Basel, Switzerland; Department of Neurology and Stroke Centre, University Hospital Basel and University of Basel, Basel, Switzerland; Department of Neurology and Stroke Centre, University Hospital Basel and University of Basel, Basel, Switzerland; Department of Imaging and Interventional Radiology, Prince of Wales Hospital, The Chinese University of Hong Kong, Hong Kong, Hong Kong; Division of Neurology, Department of Medicine, The University of Hong Kong, Hong Kong, Hong Kong; Department of Imaging and Interventional Radiology, Prince of Wales Hospital, The Chinese University of Hong Kong, Hong Kong, Hong Kong; Department of Medicine and Therapeutics, Prince of Wales Hospital, The Chinese University of Hong Kong, Hong Kong, Hong Kong; Department of Neurology, University Hospital Inselspital Bern, University of Bern, Bern, Switzerland; Department of Neurology, University Hospital Inselspital Bern, University of Bern, Bern, Switzerland; Department of Neurology, University Hospital Inselspital Bern, University of Bern, Bern, Switzerland; Department of Neurology, Medical University of Graz, Graz, Austria; Department of Neurology, Medical University of Graz, Graz, Austria; Department of Radiology and Nuclear Medicine, Erasmus MC, Rotterdam, the Netherlands; Department of Cerebrovascular Medicine, National Cerebral and Cardiovascular Center, Suita, Osaka, Japan; Department of Neurology, University Hospital of Würzburg, Würzburg, Germany; Stroke and Ageing Research Group, School of Clinical Sciences at Monash Health, Monash University, Melbourne, Australia; Peninsula Clinical School, Peninsula Health, Monash University, Melbourne, Australia; Department of Neurology, Yonsei University College of Medicine, Seoul, South Korea; Department of Neurology, Seoul National University Bundang Hospital, Seoul National University College of Medicine, Seongnam, Republic of Korea; Department of Neurology, Kansai Medical University, Hirakata, Osaka, Japan; Department of Neurology, İstanbul Nişantaşı University, İstanbul, Turkey; Calgary Stroke Program, Department of Clinical Neurosciences, Radiology and Community Health Sciences, Hotchkiss Brain Institute, University of Calgary, Calgary, Alberta, Canada; Centre for Clinical Brain Sciences, Edinburgh Imaging, and UK Dementia Institute at the University of Edinburgh, Edinburgh, United Kingdom; Division of Neurology, Department of Medicine, The University of Hong Kong, Hong Kong, Hong Kong; Department of Neurology and Stroke Centre, University Hospital Basel and University of Basel, Basel, Switzerland; Neurology and Neurorehabilitation, University Department of Geriatric Medicine FELIX PLATTER, University of Basel, Basel Switzerland; Department of Neurology and Stroke Centre, University Hospital Basel and University of Basel, Basel, Switzerland; Stroke Center Klinik Hirslanden, Hospital Zürich, Zürich, Switzerland; Neurology and Neurorehabilitation, University Department of Geriatric Medicine FELIX PLATTER, University of Basel, Basel Switzerland; Department of Medicine and Therapeutics, Prince of Wales Hospital, The Chinese University of Hong Kong, Hong Kong, Hong Kong; UCL Stroke Research Centre, Department of Translational Neuroscience and Stroke, University College London Queen Square Institute of Neurology, London, United Kingdom; Comprehensive Stroke Service, National Hospital for Neurology and Neurosurgery, University College London Hospitals NHS Trust, London, United Kingdom; Department of Renal Medicine, University College London, London, United Kingdom; UCL Stroke Research Centre, Department of Translational Neuroscience and Stroke, University College London Queen Square Institute of Neurology, London, United Kingdom; Comprehensive Stroke Service, National Hospital for Neurology and Neurosurgery, University College London Hospitals NHS Trust, London, United Kingdom; Department of Neurology, Seoul Hospital, Ewha Womans University College of Medicine, Seoul, South Korea

**Keywords:** chronic kidney disease, glomerular hyperfiltration, ischaemic stroke, mortality, recurrent stroke

## Abstract

**Introduction:**

Glomerular hyperfiltration has previously been associated with cardiovascular events and mortality but has scarcely been investigated in patients with stroke.

**Patients and methods:**

We used pooled data from an individual patient data meta-analysis of prospective, cohort studies of stroke or TIA populations. For this analysis, we included participants from study sites that collected estimated glomerular filtration rate (eGFR) at stroke presentation. Using Cox proportional hazards regression, we investigated the risk of death, any stroke and vascular death according to glomerular hyperfiltration, defined as having an eGFR greater than the age- and sex-adjusted 95th percentile. We also investigated these outcomes according to eGFR as a continuous variable, modelled using fractional polynomials.

**Results:**

A total of 11,175 patients (mean age 70.7 years, 42% female) were included in the analysis, 554 (4.9%) with hyperfiltration. Compared to the normofiltration group (absence of hyperfiltration and eGFR ≥ 60 mL/min/1.73 m^2^), the hyperfiltration group had a higher rate of all-cause death, 147 per 1000 person-years (95% CI, 119–180) vs 61 (95% CI, 57–66). Compared to normofiltration, hyperfiltration was independently associated with the risk of death from any cause (adjusted hazard ratio [HR] 1.76; 95% CI, 1.46–2.11; *P* < .001) and the risk of vascular death (adjusted HR 1.68; 95% CI, 1.29–2.17; *P* < .001). There were non-linear associations of eGFR with risk of death and vascular death, with increasing risk at both low and high eGFR (*P*_non-linearity_ < .001 for both).

**Discussion and conclusion:**

Glomerular hyperfiltration was associated with a 76% increased risk of death and a 68% increased risk of vascular death in multivariable models adjusted for age, sex and comorbidities. Glomerular hyperfiltration may be associated with adverse health outcomes, specifically in patients with ischaemic stroke. Further research is needed to confirm these findings.

## Graphical Abstract

**Graphical Abstract:**
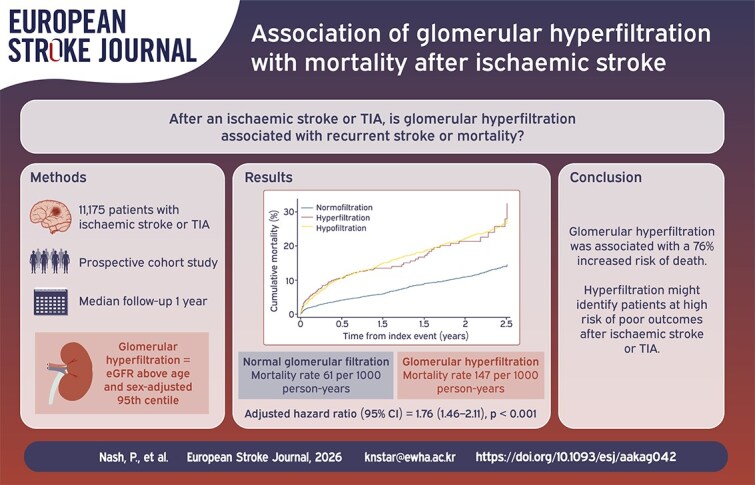

## Introduction

Chronic kidney disease (CKD) is associated with cerebrovascular and cardiovascular disease and their risk factors such as cerebral small vessel disease, hypertension, diabetes, atrial fibrillation, myocardial infarction and heart failure.^1,2^ A key indicator of CKD is a diminished estimated glomerular filtration rate (eGFR), and decreased eGFR levels have a significant association with the likelihood of developing cardiovascular disease.^1,3^ In stroke populations, impaired kidney function is closely related to stroke occurrence and poor outcomes.^4^ These consistent associations can be attributed to the shared vascular risk factors between impaired kidney function and stroke, such as hypertension, diabetes mellitus and atherosclerosis. The presence of impaired kidney function is known to exacerbate the pathophysiological processes underlying stroke, including endothelial dysfunction, oxidative stress and inflammation.^4^

Glomerular hyperfiltration has been defined as a pathologically increased single nephron GFR in response to reduced nephron mass.^5^ It is an early marker of glomerular diseases such as diabetic nephropathy^6^ and obesity-associated focal segmental glomerular sclerosis,^7^ and increased eGFR has been associated with subclinical cardiovascular disease in subjects without diabetes or albuminuria.^8^ After adjusting for age, glomerular hyperfiltration has been shown to be associated with adverse cardiovascular outcomes in those with atherosclerotic vascular disease and asymptomatic individuals.^9,10^ It has also recently been associated with an increased risk of cardiovascular disease^11^ and death^12^ in those with diabetes.

In a recent study, both high and low eGFR were independently associated with 6-month mortality after reperfusion therapy in patients with stroke.^13^ However, there has been little further research to date on the significance of glomerular hyperfiltration in stroke populations. We recently used pooled data from the Microbleeds International Collaborative Network (MICON) to demonstrate an independent association of impaired kidney function with the risk of recurrent stroke.^14^ For the current study, we aimed to investigate associations of glomerular hyperfiltration with vascular outcomes, including recurrent stroke, death and vascular death, in a large multi-centre international population of patients with stroke and TIA. We hypothesised that glomerular hyperfiltration would be associated with poor vascular outcomes, including mortality after ischaemic stroke (IS).

## Patients and methods

This study is reported according to the Strengthening the Reporting of Observational Studies in Epidemiology (STROBE) recommendations.

### Subjects

The design and inception of the MICON have been described previously.^15^ Briefly, suitable prospective cohort studies of patients with stroke or TIA, collecting data on microbleeds and recurrent stroke for at least 3 months and up to 5 years, were identified by a systematic literature search, recruiting between 2000 and 2018. The authors of eligible studies were invited to contribute data to an individual patient data meta-analysis. Thirty-eight study groups joined the collaboration and contributed data. Each study performed its own neuroimaging analyses according to local study protocols. For this study, we invited the original MICON collaboration authors to contribute data on eGFR. The MICON collaboration database contains individual patient data across a diverse range of geographic regions, including Europe, North America, the Middle East, Asia and Australia. All included patients underwent MRI. A critical aspect of these MRI examinations was the evaluation of cerebral microbleeds. Moreover, the database required detailed information on the antithrombotic treatments administered to these patients, as well as comprehensive records of any subsequent cerebrovascular events and mortality data during the follow-up period. All of the studies contributing to MICON were independently assessed by 2 reviewers as having a low risk of selection bias, using the Cochrane Collaboration tool,^16^ since they enrolled consecutive or near-consecutive eligible patients.^15^

For this study, we included the following information from the MICON database: demographic data, stroke subtypes based on Trial of ORG 10172 in Acute Stroke Treatment (TOAST) classification,^17^ vascular risk factors including previous hypertension, atrial fibrillation, diabetes, hyperlipidaemia, current smoking, stroke history, brain MRI findings and laboratory findings including eGFR, taken within 24 h of index hospitalisation, and before any reperfusion therapies. Since data on kidney function were added to the database retrospectively, it was not possible to standardise creatinine assays across centres.

### Patient selection

Patients were eligible for inclusion in the study if they were part of the original MICON meta-analysis and had data available on kidney function. Exclusion criteria included: no outcome data available; no kidney function data available and missing demographic data needed to determine eGFR.

### Estimation of glomerular filtration rate

In this international collaborative study, we chose to use the original 2009 Chronic Kidney Disease Epidemiology Collaboration (CKD-EPI) equation,^18^ without including the ethnicity coefficient, to estimate eGFR. A very large international consortium of studies has validated the 2009 equation for use in a significant majority of populations.^19^ The study authors demonstrated that the equation more accurately predicted significant clinical events (eg, commencing renal replacement therapy or adverse cardiovascular outcomes) than the widely used Modified Diet in Renal Disease equation,^20^ using data from 1.1 million participants from 16 countries covering 4 continents. Over 70% of the participants came from Asian populations. There are numerous variations of the main eGFR equations for Asian populations,^21,22^ but we are unaware of high-quality validation studies of these equations.

While Kidney Disease Improving Global Outcomes (KDIGO) has recently recommended using the updated 2021 CKD-EPI equation (which removes the ethnicity coefficient from the original equation and adjusts for its omission),^23^ we chose not to use it because, in European populations, compared to the original 2009 equation, this equation has recently been shown not to be as accurate compared to measured GFR,^24^ nor to predict clinical outcomes as well,^25^ and the European Renal Association has not recommended its adoption in Europe.^26^ This position is also supported by a consensus statement published by the European Federation of Clinical Chemistry and Laboratory Medicine.^27^

Glomerular hyperfiltration was defined as having an eGFR greater than the 95th percentile adjusted for age, sex and East Asian study centre.^28,29^ We determined the eGFR cut-offs using a quantile regression model adjusting for age and stratified by sex and East Asian study centre. We plotted the predicted cut-offs against age (Figure S1) to ensure that the model gave reasonable values. Glomerular hypofiltration was defined as eGFR < 60 mL/min/1.73 m^2^, as recommended by KDIGO. The eGFR measurement was based on laboratory findings performed at the time of admission for stroke or TIA.

**Figure 1:**
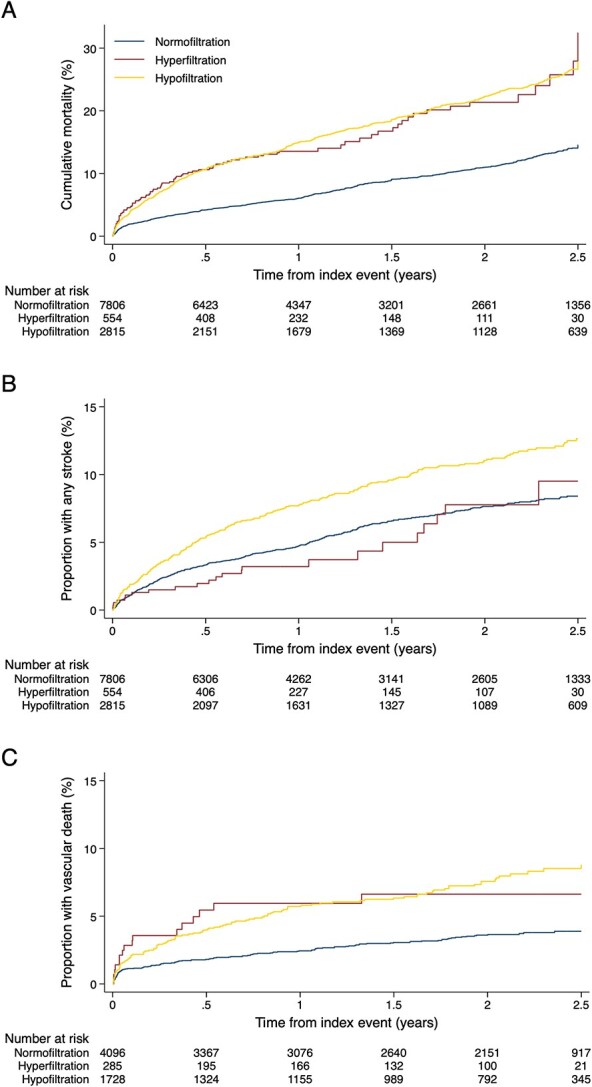
Kaplan–Meier event-rate plots showing, according to renal filtration category: (A) death from any cause; (B) composite of recurrent ischaemic stroke and symptomatic ICH; (C) vascular death.

### Outcomes

We investigated associations of filtration category (normofiltration, hyperfiltration and hypofiltration) with the risk of death from any cause, any stroke (a composite of recurrent IS and symptomatic ICH) and vascular death (defined as death from IS, ICH, myocardial infarction, heart failure or sudden death). These outcomes were prospectively collected and adjudicated by local investigators. When investigating vascular death, we first excluded sites that did not collect that outcome.

### Statistical analysis

For numerical variables, we examined histogram plots to determine their distributions and presented results as mean (SD) or median (IQR). We presented categorical variables as numbers and percentages. To assess potential sources of bias caused by loss to follow-up and our selection criteria, we compared the characteristics of included and excluded participants.

To investigate the association of glomerular filtration with the incidence of vascular outcomes, we first stratified participants into glomerular filtration categories (normofiltration, hyperfiltration and hypofiltration). We quantified the occurrence of outcome events in each group by constructing Kaplan–Meier event rate curves, and differences between glomerular filtration categories were assessed through log-rank tests. Hazard ratios (HRs) and 95% CIs for the association between glomerular filtration category and vascular outcome were estimated by fitting Cox proportional hazard models with the normofiltration group as the reference group. We assessed the proportional hazards assumption by inspecting log–log plots, and if there were concerns visually, we tested the assumption with post-estimation significance tests. Secondly, we investigated the risk of outcomes according to eGFR as a continuous variable, modelled using fractional polynomials to account for potential non-linearity in the data.

Multivariable Cox regression analysis was employed to adjust for potential confounding factors, selected according to existing evidence and biological plausibility. We included all variables in all models regardless of the *P* values in univariable analyses, based on existing data and clinical experience. In line with the STROBE statement, we did not select variables for inclusion in multivariable models on the basis of univariable *P* values.^30^ Information on a history of heart failure and alcohol intake was available for too few patients in the MICON dataset to allow reliable adjustment; therefore, these variables were not included in the multivariable analyses. The prespecified covariates (percentage data completeness) were age (100%), sex (100%), East Asian study centre (100%), atrial fibrillation (99%), hypertension (100%), diabetes (98%), hyperlipidaemia (98%), previous stroke (either IS or ICH, 100%), ischaemic heart disease (96%), current smoker (94%) and presentation with IS (rather than TIA, 100%). When investigating any stroke as the outcome, we additionally adjusted for cerebral microbleed presence, and we fitted an additional model adjusting for index stroke aetiology according to the TOAST classification. We did not report this as a main result as several centres did not collect this variable, losing over 1200 observations from the analysis. We adjusted for clustering within study centres using cluster-robust variance estimators. If confounding variables violated the proportional hazards assumption, we repeated the analysis stratifying for these variables. If the significance tests indicated that the proportional hazards assumption might be violated for the predictor variable of interest (filtration category), we inspected plots of the residuals and fitted Cox models with a time-varying coefficient to estimate the degree to which this might influence our results. To account for the potential competing risk of mortality, we also fitted competing risk regression models for any stroke and vascular death as outcomes, using the same dependent and independent variables as above. For any stroke, the competing risk was all-cause mortality, and for vascular death, non-vascular death was the competing risk. We accounted for missing data using multiple imputation with chained equations (50 equations). This was not needed for clinical outcomes or eGFR. We assessed between-centre heterogeneity by fitting interaction terms between filtration category and centre.

### Sensitivity and subgroup analyses

Since we did not have data on index stroke severity, an important predictor of early death after stroke, we explored the impact of this by conducting 2 sensitivity analyses: the first including only those patients known to have survived at least 90 days; and the second additionally adjusting for index stroke aetiology according to the TOAST classification,^17^ a proxy for stroke severity. We further investigated this by conducting subgroup analyses according to factors known to be associated with stroke severity—age, TOAST classification, atrial fibrillation and heart failure. For ease of interpretation and presentation of these subgroup analyses, we excluded patients with hypofiltration (eGFR < 60 mL/min/1.73 m^2^) and compared those with hyperfiltration to those with normal glomerular filtration (*n* = 554 and *n* = 7806, respectively). We fitted separate Cox regression models with interaction terms between hyperfiltration and each of the subgroups listed above, including the same covariates as the models used for the main results.

Although the CKD Prognosis Consortium provides robust evidence that the CKD-EPI 2009 equation accurately predicts outcomes for both Eastern and Western populations, this paper concerned reduced eGFR and CKD rather than hyperfiltration. Therefore, we carried out 2 further sensitivity analyses to assess the impact of any possible measurement bias causing misclassification of filtration category. The first used the 2021 CKD-EPI equation to determine eGFR, and the second used eGFR equations developed within participating countries, where available. The equations used were the Japanese Society of Nephrology equation^31^ for Japanese stroke populations, the Korean Full Age Spectrum (FAS) equation^32^ for South Korean centres, and the CKD-EPI equation using adapted coefficients for Chinese populations.^33^ We continued to use the CKD-EPI 2009 equation for European and North American centres.

Statistical analyses were performed using Stata (version 18.1, StataCorp), and significance was established with a 2-sided *P*-value less than .05.

### Standard protocol approvals, registrations and patient consents

The research received authorisation from the UK Health Research Authority (reference number 8/HRA/0188). Each of the included cohort studies had previously secured the necessary ethical and regulatory approvals as per their respective local guidelines. The data shared for this pooled analysis of individual patient data were completely anonymised, ensuring that the identification of individual participants was not possible. Consequently, the need to obtain specific consent from each participant for this analysis was waived by the research ethics committee.

## Results

Eighteen of the original MICON collaborating groups contributed data to this study, providing 11,660 participants to consider for eligibility, of whom 485 were excluded. The reasons for exclusion included missing kidney function or outcome data, and missing baseline characteristics that prevented calculation of eGFR. For full details, see the study selection flowchart (Figure S1). Compared to those included in the study, the excluded group had a slightly higher rate of female participants, higher rates of ischaemic heart disease and antiplatelet use and a lower proportion of patients with reduced eGFR (Table S1).

We included 11,175 patients (mean age 70.7 years, 42.3% female) in the analysis, 554 (4.9%) with glomerular hyperfiltration and 2815 (25.1%) with hypofiltration (eGFR < 60 mL/min/1.73 m^2^). The hyperfiltration group was slightly older than the normofiltration group, and considerably younger than the hypofiltration group (mean age 70.7 ± 14.1 years compared to 68.3 ± 12.6 for the normofiltration group and 77.3 ± 9.7 for the hypofiltration group). The respective mean (SD) and median (IQR) eGFRs were: 83.9 (13.8) and 84 (73–93) for normofiltration; 104.3 (14.4) and 102 (93–113) for hyperfiltration and 44.7 (12.8) and 48 (38–55) for hypofiltration. The eGFR distribution according to filtration category is shown in Figure S3. Compared to the normofiltration group, there were higher rates of atrial fibrillation (48.9% vs 44.0%), heart failure (13.8% vs 9.3%) and cardioembolic stroke (47.7% vs 43.8%) in the hyperfiltration group, but otherwise the cardiovascular risk factors were balanced between the 2 groups (Table 1). Apart from smoking, all cardiovascular risk factors were more prevalent in the hypofiltration group than in the other groups. Small vessel disease markers according to glomerular filtration are shown in Table S2. Compared to normofiltration, there were higher rates of severe periventricular white matter hyperintensities (Fazekas scale 2–3) in the hyperfiltration group (49.4% vs 29.7%). The rates of cerebral microbleed presence and lacune presence were similar (26.4% vs 28.5% and 29.0% vs 27.9%, respectively, Table S2).

**Table 1:** Baseline characteristics according to glomerular filtration.

	Normofiltration (*n* = 7806)	Hyperfiltration (*n* = 554)	Hypofiltration (*n* = 2815)
**Clinical data**			
** Age; years; mean (SD)**	68.3 (12.6)	70.7 (14.1)	77.3 (9.7)
** Female sex; *n***	3036 (38.9%)	234 (42.2%)	1452 (51.6%)
** East Asian study centre**	4903 (62.8%)	331 (59.7%)	1439 (51.1%)
** Atrial fibrillation**	3410 (44.0%)	267 (48.9%)	1741 (62.1%)
** Hypertension**	5297 (68.0%)	377 (68.2%)	2345 (83.6%)
** Diabetes**	1726 (22.6%)	133 (24.4%)	880 (31.7%)
** Hyperlipidaemia**	2775 (36.4%)	173 (31.7%)	1208 (43.5%)
** Previous ischaemic stroke**	1022 (13.1%)	85 (15.3%)	525 (18.7%)
** Previous ICH**	102 (1.3%)	9 (1.7%)	56 (2.0%)
** Ischaemic heart disease**	843 (11.3%)	55 (10.3%)	565 (20.7%)
** Heart failure**	326 (9.3%)	30 (13.8%)	250 (15.7%)
** Current smoker**	1521 (20.7%)	108 (21.3%)	326 (12.3%)
** Alcohol use**	625 (21.4%)	39 (18.8%)	258 (16.9%)
** eGFR; mean (SD)**	83.9 (13.8)	104.3 (14.4)	44.7 (12.8)
** eGFR; median (IQR)**	84 (73–93)	102 (93–113)	48 (38–55)
** eGFR; range**	60–143	74–153	2–60
**Presentation**			
** Transient ischaemic attack**	965 (12.4%)	52 (9.4%)	314 (11.2%)
** Ischaemic stroke**	6841 (87.6%)	502 (90.6%)	2501 (88.8%)
**TOAST classification**			
** Large artery atherosclerosis**	1335 (20.5%)	99 (22.3%)	341 (14.7%)
** Cardioembolic**	2858 (43.8%)	212 (47.7%)	1285 (55.3%)
** Small vessel disease**	1038 (15.9%)	50 (11.3%)	254 (10.9%)
** Other known cause**	286 (4.4%)	13 (2.9%)	68 (2.9%)
** Unknown cause**	1005 (15.4%)	70 (15.8%)	374 (16.1%)
**Neuroimaging data**			
** Cerebral microbleeds present**	2222 (28.5%)	146 (26.4%)	947 (33.6%)
**CMB severity category**			
** 0**	5584 (71.5%)	408 (73.6%)	1868 (66.4%)
** 1**	1009 (12.9%)	56 (10.1%)	357 (12.7%)
** 2–4**	761 (9.7%)	57 (10.3%)	357 (12.7%)
** >5**	452 (5.8%)	33 (6.0%)	233 (8.3%)
**Microbleed distribution**			
** None**	5584 (71.5%)	408 (73.6%)	1868 (66.4%)
** Strictly lobar CMB**	643 (8.2%)	36 (6.5%)	314 (11.2%)
** Strictly deep CMB**	828 (10.6%)	55 (9.9%)	278 (9.9%)
** Mixed CMB**	649 (8.3%)	44 (7.9%)	304 (10.8%)
** CMB distribution unknown**	102 (1.3%)	11 (2.0%)	51 (1.8%)

Abbreviations: eGFR = estimated glomerular filtration rate; CMB = cerebral microbleeds; TOAST = Trial of ORG 10172 in Acute Stroke Treatment.

### Outcome events during follow-up

Over a median follow-up period of 1.0 year (IQR 0.8–2.1), there were 1533 deaths from any cause, at a rate of 84 per 1000 patient-years (95% CI, 80–89), and 802 recurrent stroke events (a composite of recurrent IS and symptomatic ICH), at a rate of 45 (95% CI, 42–48) per 1000 patient-years (Figure 1, Table 2). Compared to the normofiltration group, the hyperfiltration group had higher rates of death, 147 (95% CI, 119–180) vs 61 (95% CI, 57–66) and vascular death, 45 (95% CI, 26–71) vs 17 (14–20; Figure 1, Table 2). There were similar event rates for any stroke (42 vs 40), recurrent IS (37 vs 35) and ICH (8 vs 7; Table S3). Log–log plots and formal tests indicated that the proportional hazards assumption was not valid for the hypofiltration group for some of the models. However, the results from stratified and time-varying regression models suggest that this does not change the conclusions (Tables S4 and S5).

**Table 2:** Recurrent stroke, death and vascular death according to glomerular filtration.

	Events (%)	Event rate per 1000 patient-years (95% CI)	Absolute rate increase per 1000 patient-years (95% CI)	Adjusted hazard ratio (95% CI)
**Death from any cause**				
**Whole cohort**	1533 (13.7)	84 (80–89)	–	–
**Normofiltration**	771 (9.8)	61 (57–66)	Ref.	Ref.
**Hyperfiltration**	93 (16.8)	147 (119–180)	86 (62–114)	1.76 (1.46–2.11)^a^
**Hypofiltration**	669 (23.8)	135 (125–146)	74 (68–80)	1.45 (1.31–1.60)^a^
**Any stroke**				
**Whole cohort**	802 (7.2)	45 (42–48)	–	–
**Normofiltration**	499 (6.4)	40 (37–43)	Ref.	Ref.
**Hyperfiltration**	26 (4.7)	42 (27–61)	1 (−10–15)	0.90 (0.53–1.53)^a^
**Hypofiltration**	277 (9.8)	58 (51–65)	17 (14–21)	1.30 (1.15–1.48)^a^
**Normofiltration**				Ref.
**Hyperfiltration**				0.75 (0.42–1.34)^b^
**Hypofiltration**				1.31 (1.15–1.49)^b^
**Vascular death** ^**c**^				
**Whole cohort**	267 (5.6)	25 (22–28)	–	–
**Normofiltration**	129 (4.0)	17 (14–20)	Ref.	Ref.
**Hyperfiltration**	17 (7.4)	45 (26–71)	27 (12–51)	1.68 (1.29–2.17)
**Hypofiltration**	121 (9.2)	43 (36–51)	26 (22–31)	1.59 (1.28–1.99)

^a^Cox regression models adjusted for age, sex, East Asian study centre, atrial fibrillation, hypertension, diabetes, hyperlipidaemia, previous stroke (IS or ICH), ischaemic heart disease, current smoker and presentation with ischaemic stroke (IS) (rather than TIA). Cerebral microbleed presence (for any stroke only); shared frailty term to account for clustering within centres.

^b^Additionally adjusted for Trial of ORG 10172 in Acute Stroke Treatment (TOAST) classification of the index stroke; 5 centres (1230 observations) excluded from the analysis

^c^Five sites did not collect data on vascular death, leaving 6109 participants for this analysis

**Figure 2:**
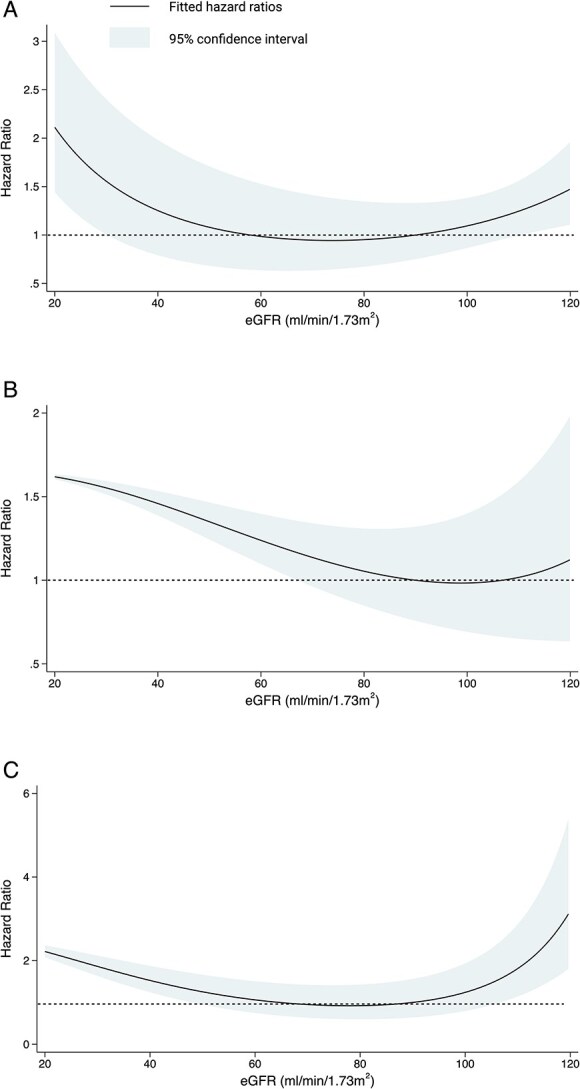
Fitted hazard ratios showing risk of (A) all-cause mortality, (B) recurrent stroke and (C) vascular death according to eGFR as a continuous variable, using a fractional polynomial Cox regression model. Abbreviation: eGFR = estimated glomerular filtration rate.

In multivariable Cox regression models, compared to normofiltration, hyperfiltration was independently associated with the risk of death from any cause (adjusted HR 1.76; 95% CI, 1.46–2.11; *P* < .001; Tables 2 and 3; Figure 2) and the risk of vascular death (adjusted HR 1.68; 95% CI, 1.29–2.17, *P* < .001). There was no evidence of an interaction between study centre and filtration category (interaction *P* = .151). There was no significant association of hyperfiltration with the risk of composite stroke events (adjusted HR 0.90; 95% CI, 0.53–1.53, *P* = .69 for the pairwise comparison between hyperfiltration and normofiltration), recurrent IS (adjusted HR 0.97; 95% CI, 0.62–1.49; Table S3) and ICH (adjusted HR 0.96; 95% CI, 0.39–2.39). In a Cox regression model additionally adjusting for TOAST classification of the index stroke, there was also no statistically significant association of hyperfiltration with any stroke (adjusted HR 0.75; 95% CI, 0.42–1.34). In competing risk regression models, there were no significant changes in the estimates. Compared to normofiltration, there was an increased risk of vascular death (adjusted HR 1.62; 95% CI, 1.25–2.10) and no association with any stroke (adjusted HR 0.89; 95% CI, 0.51–1.54).

**Table 3:** Multivariable Cox regression models predicting risk of death, recurrent stroke and vascular death according to glomerular filtration.

	All-cause mortality	Recurrent stroke^a^	Vascular death
Predictor	Adjusted HR (95% CI)	Adjusted HR (95% CI)	Adjusted HR (95% CI)
**Glomerular filtration**			
**Normofiltration**	Ref.	Ref.	Ref.
**Hyperfiltration**	1.76 (1.46–2.11)	0.90 (0.53–1.53)	1.68 (1.29–2.17)
**Hypofiltration**	1.45 (1.31–1.60)	1.30 (1.15–1.48)	1.59 (1.28–1.99)
**Age**	1.07 (1.06–1.08)	1.01 (1.00–1.02)	1.06 (1.03–1.08)
**Sex; female**	0.94 (0.82–1.08)	1.15 (0.99–1.34)	1.36 (1.04–1.78)
**East Asian study centre**	1.59 (1.03–2.45)	0.95 (0.64–1.44)	1.33 (0.74–2.39)
**Atrial fibrillation**	1.37 (0.89–2.11)	0.73 (0.52–1.02)	1.36 (0.91–2.03)
**Hypertension**	0.91 (0.78–1.06)	1.11 (0.90–1.36)	0.82 (0.59–1.13)
**Diabetes**	1.39 (1.22–1.59)	1.11 (0.90–1.37)	1.73 (1.43–2.09)
**Hyperlipidaemia**	0.83 (0.73–0.95)	1.03 (0.87–1.21)	0.92 (0.76–1.11)
**Previous stroke (IS or ICH)**	1.27 (1.10–1.47)	1.90 (1.56–2.31)	1.18 (0.81–1.71)
**Ischaemic heart disease**	1.29 (1.14–1.45)	1.18 (0.98–1.43)	1.44 (1.24–1.66)
**Current smoker**	1.16 (0.92–1.49)	0.97 (0.77–1.21)	1.25 (0.86–1.83)
**Presentation with ischaemic stroke**	2.35 (1.54–3.58)	1.34 (0.97–1.86)	2.38 (1.36–4.17)
**Current smoker**	1.16 (0.92–1.49)	0.97 (0.77–1.21)	1.25 (0.86–1.83)
**Microbleeds present**	–	1.26 (1.05–1.50)	–

Abbreviations: HR = hazard ratio; IS = ischaemic stroke.

Clustering within study centres adjusted for using cluster-robust variance estimators

^a^A composite of ischaemic stroke or ICH.

Fractional polynomial Cox regression demonstrated non-linear associations of eGFR with the risks of death and vascular death (*P* for non-linearity < .001 for both outcomes) with higher hazard ratios at low and high eGFR (Figure 2). There was no statistically significant non-linearity for any stroke (*P* = .07).

**Figure 3:**
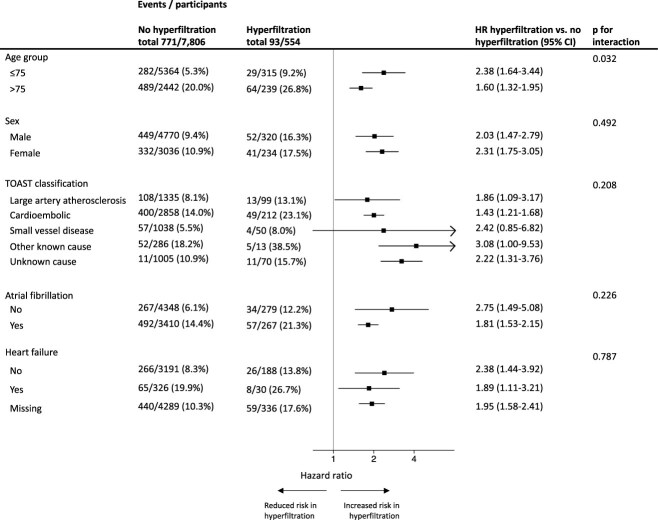
Subgroup analysis of the risk of all-cause mortality according to hyperfiltration, age group, sex, index stroke aetiology, atrial fibrillation and heart failure.

### Sensitivity analyses

To assess the possible impact of missing stroke severity data, in the sensitivity analysis including only cases known to survive for at least 90 days of follow-up, compared to normofiltration, there remained an increased risk of all-cause mortality for those with glomerular hyperfiltration and hypofiltration. The adjusted HRs were 1.46 (95% CI, 1.13–1.88) and 1.35 (95% CI, 1.16–1.56) for hyperfiltration and hypofiltration, respectively. In the analysis including the TOAST category in the adjusted models the respective adjusted HRs were 1.77 (95% CI, 1.44–2.16) and 1.46 (95% CI, 1.35–1.59).

In the sensitivity analyses to assess the possible impact of measurement bias caused by different methods of estimating GFR, there was no evidence that different methods affected the results. In the analysis using the 2021 CKD-EPI equation, compared to normofiltration, the respective adjusted HRs for hyperfiltration and hypofiltration were 1.63 (95% CI, 1.32–2.01) and 1.42 (95% CI, 1.23–1.64). In the analysis using locally developed eGFR equations, the respective adjusted HRs for hyperfiltration and hypofiltration were 1.74 (95% CI, 1.40–2.14) and 1.62 (95% CI, 1.27–2.07).

### Subgroup analyses

We found little heterogeneity of the risks of all-cause mortality and vascular death according to hyperfiltration in subgroups of age, sex, index stroke aetiology, atrial fibrillation and heart failure. Owing to small numbers of events in some TOAST subgroups, it was not possible to conduct a meaningful subgroup analysis for vascular death according to index stroke aetiology. Full details are shown in Figures 3 and S4.

## Discussion

In this large multinational, pooled individual patient-data analysis, we demonstrated that glomerular hyperfiltration was independently associated with the risk of death, and the risk of vascular death. The absolute event rate was more than 2.5 times higher in the glomerular hyperfiltration group than in the normal filtration group for both all-cause mortality (147 vs 61 deaths per 1000 patient-years) and vascular death (27 vs 11). This was despite similar rates of most cardiovascular risk factors in the hyperfiltration and normofiltration groups, indicating that previously unexplored mechanisms might be responsible for this effect. The increased risk of death and vascular death in glomerular filtration was also demonstrated in Cox regression models including eGFR as a continuous variable, with evidence of non-linearity with increasing risk at both low and high eGFR. Our findings were robust to sensitivity analyses assessing the impact of unknown stroke severity and the use of different eGFR equations, and consistent findings were seen in subgroups known to be associated with both severe (eg, cardioembolic) and minor stroke (eg, small vessel occlusion). In contrast, there was no association between hyperfiltration and recurrent stroke in our population.

Glomerular hyperfiltration is considered a clinical marker of early kidney impairment, especially in individuals with diabetes and hypertension.^34,35^ The phenomenon of glomerular hyperfiltration can be caused by afferent arteriolar vasodilation or by efferent arteriolar vasoconstriction owing to overactivation of the renin–angiotensin–aldosterone system.^36^ In a study monitoring 502 patients with a history of hypertension, glomerular hyperfiltration was an independent predictor of urinary albumin excretion after 8 years of follow-up.^34^ Moreover, a previous study of a Japanese population with prediabetes and prehypertension showed that hyperfiltration was associated with the progression to clinical diabetes and hypertension.^28^ Glomerular hyperfiltration, demonstrating an association with components of metabolic syndrome,^37^ has been recognised as an independent predictor of cardiovascular outcomes. According to a recent study that followed 8794 middle-aged or elderly patients for 6.2 years, eGFR and adverse cardiovascular outcomes had a U-shaped relationship.^38^ In addition, a retrospective study of approximately 43,500 individuals from a general population showed an association of hyperfiltration with an increased risk of cardiovascular-related mortality after adjustment for sex, age, muscle mass and history of hypertension and diabetes mellitus.^39^ Our findings are in line with the results of these previous studies, providing additional information that glomerular hyperfiltration is also associated with mortality and vascular mortality in patients with stroke.

Although our study does not demonstrate a definite mechanism underlying this association, the excess risk of death in the hyperfiltration group is likely to be mediated in part by cardiovascular causes, and the lack of association with stroke during follow-up suggests coronary or heart failure events. Indeed, glomerular hyperfiltration was associated with vascular death in our cohort, but we did not have complete data on specific causes of death, so we cannot be certain that the excess deaths in the hyperfiltration group were cardiac. Nonetheless, since we found a high risk of mortality and vascular death for those with glomerular hyperfiltration, but no increased risk of recurrent stroke, our results suggest that hyperfiltration identifies a group of patients with high cardiovascular vulnerability who need careful medical management. There are other possible plausible mechanisms for this increased risk. Glomerular hyperfiltration is associated with dysfunction in the renin-angiotensin–aldosterone axis, vascular inflammation, arterial stiffness and endothelial dysfunction, all of which are associated with cardiovascular disease and a higher risk of death.^38,40,41^ This may lead to glomerular hypertension and potentially glomerular hypertrophy, albuminuria and reduced eGFR and it may contribute to increased mortality in patients after stroke.^6,42,43^ These changes might be both a cause and a consequence of renal injury. A high glomerular filtration rate was related to impaired arterial stiffness investigated by the augmentation index.^44^ A previous study showed that the augmentation index was associated with in-hospital mortality in patients with acute IS.^45^ Although our dataset did not contain arterial stiffness-related markers, this relationship of arterial stiffness with stroke outcome could be proposed as a possible hypothesis for our findings. Endothelial dysfunction is an important predictor of poor cardiovascular outcome in vascular diseases, and it is also associated with a poor prognosis in patients with stroke.^46^ Glomerular hyperfiltration may lead to endothelial dysfunction, and therefore may partly explain our findings.^42,43^ Further study is needed on the mechanisms of the association between glomerular hyperfiltration and prognosis, particularly including mortality, in patients with stroke. In contrast, in our study, glomerular hyperfiltration was not associated with the risk of recurrent stroke. The absence of a clear association between glomerular hyperfiltration and recurrent stroke in our analysis should be interpreted in the context of a high-risk secondary prevention population, in which all patients already have established cerebrovascular disease and are receiving intensive vascular risk management. In this setting, recurrent stroke risk is likely dominated by factors such as stroke mechanism, atherosclerotic burden, atrial fibrillation and secondary preventive treatment, so glomerular hyperfiltration may not confer a substantial additional independent effect on recurrence in this high-risk stroke population.

It is possible that in our population recurrent stroke events in the hyperfiltration group might have had a higher mortality, although we have not been able to demonstrate this with our data. Compared to the normofiltration group, there was a higher rate of cardioembolism as the aetiology of the index stroke in the hyperfiltration group, a stroke subtype that is associated with more severe stroke. Endothelial dysfunction could cause worse collateral blood flow, possibly leading to higher infarct volumes and more cerebral swelling. An association of high eGFR and moderate-to-severe stroke severity was found in a study from the Korean Stroke Registry,^47^ but this needs to be confirmed in further studies.

From a clinical perspective, our findings raise the hypothesis that glomerular hyperfiltration may identify a subgroup of patients with stroke who have a particularly adverse vascular and renal risk profile. In other clinical settings, therapies that modulate intraglomerular haemodynamics and mitigate hyperfiltration physiology have been associated with renoprotective effects and slower progression of CKD.^48–50^ A mechanistic randomised controlled trial of sodium-glucose cotransporter-2 (SGLT2) inhibitors as an add-on therapy to angiotensin-converting enzyme inhibitors showed an additional reduction in measured GFR of 8 mL/min/1.73 m^2^ in the treatment group after 8 weeks, along with an additional systolic blood pressure lowering of 4 mmHg, in patients with type 1 diabetes and glomerular hyperfiltration.^49^ It is possible that alleviation of hyperfiltration might contribute to the mechanisms behind the known improvements in cardiovascular risk provided by sodium glucose cotransporter-2 inhibitors for patients with diabetes,^51,52^ heart failure^53,54^ and albuminuric CKD.^48,50^

### Strengths and limitations

Our study has several strengths, including its large sample size and extensive geographical reach, increasing the generalisability of the findings. Each cohort contributing to the individual meta-analysis was prospective in design. We compared clinical outcomes according to glomerular filtration using both numerical and categorical methods with consistent results. There are some limitations to our study. First, although we made considerable efforts to adjust for potential confounders in our analysis, the observational nature of the study design introduces the possibility of residual confounding, and means that we were unable to demonstrate a causative link between hyperfiltration and outcomes. Rather we have demonstrated that hyperfiltration identifies a group of patients at high risk of mortality after stroke, suggesting that it should be interpreted as a marker of systemic cardiorenal and vascular vulnerability. However, given the strength of our observed association of hyperfiltration with mortality, consistent findings across multiple subgroups and sensitivity analyses, and the plausible potential pathophysiological mechanisms, a causal link is possible; future longitudinal and interventional studies are needed to test this hypothesis. Second, our definition of glomerular hyperfiltration will have included some patients with normal GFR, since it is not possible to determine single nephron GFR without invasive tests. Nonetheless, as our definition identified a group at risk of poor outcomes, it is likely that a significant proportion of the glomerular hyperfiltration group in our study truly had pathologically increased single nephron GFR. Third, because of a lack of a harmonised measure of baseline stroke severity (eg, NIHSS) across contributing cohorts, residual confounding by stroke severity cannot be excluded, particularly for mortality outcomes, and the direction of this confounding might be to reduce the magnitude of our observed association. Stroke severity could also potentially be a mediator of our findings. Nevertheless, our findings remained robust in sensitivity analyses (excluding deaths before 90 days and including TOAST classification as a covariate) and subgroup analyses (defined by age, stroke aetiology, atrial fibrillation and heart failure, factors known to be associated with stroke severity), showing consistently increased mortality risk in those with hyperfiltration. Indeed, even if the increased mortality for those with glomerular hyperfiltration were mediated primarily through stroke severity (which is unknown), this is still of scientific interest. However, since increased risks of death and cardiovascular disease have been observed in multiple populations without stroke,^8–12^ it is likely that other mechanisms are contributing to this risk. Fourth, we only had access to a single measurement of kidney function, so we were unable to account for any potential inaccuracies caused by acute kidney injury (AKI). Some patients with hyperfiltration will have been misclassified as normofiltration or hypofiltration because of missed AKI. However, since the physiological stresses associated with acute stroke (eg, sepsis, volume depletion) would mainly cause reduction in kidney function, they are unlikely to cause increased GFR, so whilst acute stressor events might reduce the sensitivity of our hyperfiltration definition (ie, meaning we miss some cases), they are unlikely to reduce the specificity. Fifth, we did not have data on reperfusion therapies, so we were unable to determine whether differential access to acute stroke treatments, or intravenous contrast administration, according to filtration category might have an effect. Finally, there were missing data on factors that could affect the risk of recurrent vascular events, including body mass index, albuminuria, adherence to antithrombotic therapy and the monitoring and control of blood pressure, including the use of renin–angiotensin–aldosterone system inhibitors. The lack of data on body mass index and muscle mass is of particular relevance because sarcopaenia is associated with low creatinine and possibly hyperfiltration. Potential bias introduced by this missing data is likely to overestimate the risk of mortality in the hyperfiltration group. However, our subgroup analysis demonstrated that both older and younger patients with hyperfiltration were at increased risk of mortality. Indeed, the point estimates of this subgroup analysis indicated that the risk of death according to hyperfiltration was higher for those aged under 75 than those over 75. Since sarcopaenia is much more common in older age groups,^55^ we estimate that the impact of missing data on muscle mass is likely to be small.

## Conclusion

In this large international stroke population, glomerular hyperfiltration was associated with the risk of death in models adjusted for age, sex and comorbidities. Findings were consistent in subgroups associated with severe and non-severe subtypes. As our study contributes to existing evidence that glomerular hyperfiltration may be associated with adverse health outcomes across a range of populations, including patients with stroke, further research is needed to clarify underlying mechanisms and potential therapeutic options.

## Supplementary Material

aakag042_MICON_hyperfiltration_ESJ_supplement_R1

## Data Availability

To facilitate the replication of procedures and outcomes, inquiries for anonymised data that were not included in the article will be considered from researchers with the requisite qualifications. A data-sharing agreement must be put in place before any data are shared. Written proposals will be assessed by the members of the MICON steering committee.
