# Supplementary material for: Association of glomerular hyperfiltration with mortality in stroke: an analysis using pooled individual patient data
Source: Eur Stroke J. 2026 May 11;11(5):aakag042. doi: 10.1093/esj/aakag042 (PMC13160420; doi:10.1093/esj/aakag042)
Supplement: aakag042_MICON_hyperfiltration_ESJ_supplement_R1 [file aakag042_micon_hyperfiltration_esj_supplement_r1.docx]

**Supplemental Material**

**Table of contents**

Page 1 Supplemental Table 1. Characteristics of included and excluded participants

Page 2 Supplemental Table 2. Cerebral small vessel disease markers according to glomerular filtration

Page 3 Supplemental Table 3. Multivariable Cox regression model, stratified for age, predicting risk of death according to glomerular filtration

Page 3 Supplemental Table 4. Multivariable Cox regression model predicting risk of any stroke according to glomerular filtration, including a time-varying coefficient for hypofiltration

Page 4 Supplemental Table 5. Multivariable Cox regression model, stratified by age, predicting risk of death according to glomerular filtration

Page 5 Supplemental Figure 1. Glomerular hyperfiltration cut-offs according to age

Page 5 Supplemental Figure 2. Study flowchart of patient selection

Page 6 Supplemental Figure 3. eGFR distributions according to glomerular filtration category

Page 7 Supplemental Figure 4. Subgroup analysis of the risk of vascular death according to hyperfiltration, age group, sex, atrial fibrillation and heart failure

**Supplemental Table 1.** Characteristics of included and excluded participants

|  | Included (n=11175) | Excluded (n=485) |
| --- | --- | --- |
| Age; years; mean (SD) | 70.7 (12.6) | 71.0 (12.8) |
| Sex; female; n | 4722 (42.3%) | 228 (47.8%) |
| East Asian study center | 6673 (59.7%) | 31 (6.4%) |
| eGFR <60 | 2815 (25.2%) | 63 (13.0%) |
| Atrial fibrillation | 5418 (48.8%) | 239 (52.5%) |
| Hypertension | 8019 (72.0%) | 337 (71.1%) |
| Diabetes | 2739 (25.0%) | 98 (23.5%) |
| Hyperlipidemia | 4156 (38.0%) | 229 (54.8%) |
| Previous ischemic stroke | 1632 (14.6%) | 76 (15.8%) |
| Previous ICrH | 167 (1.5%) | 3 (0.8%) |
| Ischemic heart disease | 1463 (13.6%) | 92 (19.7%) |
| Current smoker | 1955 (18.6%) | 59 (19.2%) |
| Antiplatelet use | 2346 (35.1%) | 166 (42.5%) |
| Anticoagulant use | 840 (12.5%) | 38 (9.5%) |

Abbreviations: eGFR = estimated glomerular filtration rate; ICrH = intracranial hemorrhage.

**Supplemental Table 2.** Cerebral small vessel disease markers according to glomerular filtration

|  | Normofiltration (n=7806) | Hyperfiltration (n=554) | Hypofiltration (n=2815) |
| --- | --- | --- | --- |
| Microbleeds present | 2222 (28.5%) | 146 (26.4%) | 947 (33.6%) |
| Lobar CMB present | 1292 (16.8%) | 80 (14.7%) | 618 (22.4%) |
| Deep CMB present | 1645 (21.1%) | 127 (22.9%) | 631 (22.4%) |
| Brainstem CMB present | 865 (11.1%) | 62 (11.2%) | 355 (12.6%) |
| Cerebellar CMB present | 952 (12.2%) | 75 (13.5%) | 407 (14.5%) |
| CMB category |  |  |  |
| 0 | 5584 (71.5%) | 408 (73.6%) | 1868 (66.4%) |
| 1 | 1009 (12.9%) | 56 (10.1%) | 357 (12.7%) |
| 2-4 | 761 (9.7%) | 57 (10.3%) | 357 (12.7%) |
| ≥5 | 452 (5.8%) | 33 (6.0%) | 233 (8.3%) |
| Microbleed distribution |  |  |  |
| None | 5584 (71.5%) | 408 (73.6%) | 1868 (66.4%) |
| Strictly lobar | 643 (8.2%) | 36 (6.5%) | 314 (11.2%) |
| Strictly deep | 828 (10.6%) | 55 (9.9%) | 278 (9.9%) |
| Mixed | 649 (8.3%) | 44 (7.9%) | 304 (10.8%) |
| Unknown | 102 (1.3%) | 11 (2.0%) | 51 (1.8%) |
| CSS |  |  |  |
| None | 5626 (98.4%) | 434 (97.7%) | 2007 (98.3%) |
| Focal | 86 (1.5%) | 10 (2.3%) | 33 (1.6%) |
| Disseminated | 4 (0.1%) | 0 (0%) | 1 (0.0%) |
| Fazekas DWM score |  |  |  |
| 0 | 829 (24.5%) | 44 (25.4%) | 306 (18.0%) |
| 1 | 1426 (42.1%) | 62 (35.8%) | 664 (39.1%) |
| 2 | 698 (20.6%) | 37 (21.4%) | 395 (23.2%) |
| 3 | 433 (12.8%) | 30 (17.3%) | 334 (19.7%) |
| Fazekas PV score |  |  |  |
| 0 | 1313 (38.9%) | 42 (22.3%) | 443 (27.9%) |
| 1 | 1058 (31.4%) | 53 (28.2%) | 452 (28.5%) |
| 2 | 570 (16.9%) | 48 (25.5%) | 394 (24.8%) |
| 3 | 430 (12.8%) | 45 (23.9%) | 297 (18.7%) |
| Total Fazekas; median (IQR) | 2 (1 to 3) | 2 (1 to 4) | 2 (1 to 4) |
| Lacunes present | 1369 (27.9%) | 89 (29.0%) | 570 (37.7%) |

Abbreviations: CMB = cerebral microbleeds; CSS = cortical superficial siderosis; DMW = deep white matter; PV = periventricular

**Supplemental Table 3.** Recurrent ischemic stroke and symptomatic intracranial hemorrhage according to glomerular filtration

|  | Rate per 1000 patient-years (95% CI) | Absolute rate increase per 1000 patient-years (95% CI) | Adjusted hazard ratio (95% CI) |
| --- | --- | --- | --- |
| **Recurrent ischemic stroke** |  |  |  |
| Whole cohort | 40 (37-43) |  |  |
| Normofiltration | 35 (32-39) | Ref. | Ref. |
| Hyperfiltration | 37 (23-55) | 2 (-8-17) | 0.97 (0.62-1.49) |
| Hypofiltration | 51 (45-58) | 16 (14-29) | 1.33 (1.11-1.58) |
|  |  |  |  |
| **Symptomatic intracranial hemorrhage** |  |  |  |
| Whole cohort | 7 (6-9) | - | - |
| Normofiltration | 7 (6-9) | Ref. | Ref. |
| Hyperfiltration | 8 (3-19) | 1 (-3-10) | 0.96 (0.39-2.39) |
| Hypofiltration | 8 (6-11) | 1 (0-3) | 1.04 (0.69-1.57) |

**Supplemental Table 4.** Multivariable Cox regression model predicting risk of any stroke according to glomerular filtration, including a time-varying coefficient for hypofiltration

| Predictor | HR (95% CI) |
| --- | --- |
| Filtration category |  |
| Normofiltration | Ref. |
| Hyperfiltration | 0.99 (0.55 to 1.77) |
| Hypofiltration | 1.49 (1.28 to 1.72) |
| Age | 1.009 (0.998 to 1.020) |
| Sex; female | 1.097 (0.940 to 1.281) |
| East Asian study center | 1.093 (0.688 to 1.737) |
| Atrial fibrillation | 0.763 (0.548 to 1.064) |
| Hypertension | 1.071 (0.868 to 1.323) |
| Diabetes | 1.116 (0.898 to 1.388) |
| Hyperlipidemia | 1.043 (0.872 to 1.248) |
| Ischemic heart disease | 1.170 (0.962 to 1.423) |
| Previous stroke | 1.899 (1.528 to 2.360) |
| Presentation with ischemic stroke (rather than transient ischemic attack) | 1.219 (0.887 to 1.676) |
| Current smoker | 1.007 (0.804 to 1.260) |
| Microbleeds present | 1.306 (1.094 to 1.558) |
| Including time-varying coefficient: |  |
| Hypofiltration | 0.88 (0.81 to 0.96) |

Clustering within study centres adjusted for using cluster-robust variance estimators

**Supplemental Table 5.** Multivariable Cox regression model, stratified for age, predicting risk of death according to glomerular filtration

| Predictor | Adjusted HR (95% CI) |
| --- | --- |
| Filtration category |  |
| Normofiltration | Ref. |
| Hyperfiltration | 1.68 (1.32 to 2.13) |
| Hypofiltration | 1.43 (1.26 to 1.63) |
| Age | Omitted |
| Sex; female | 0.938 (0.809 to 1.088) |
| East Asian study center | 1.789 (1.201 to 2.666) |
| Atrial fibrillation | 1.385 (0.854 to 2.246) |
| Ischemic heart disease | 1.327 (1.169 to 1.507) |
| Diabetes | 1.359 (1.186 to 1.556) |
| Hyperlipidemia | 0.823 (0.705 to 0.961) |
| Previous stroke | 1.253 (1.092 to 1.438) |
| Current smoker | 1.157 (0.925 to 1.446) |
| Presentation with ischemic stroke (rather than transient ischemic attack) | 2.284 (1.426 to 3.656) |

Clustering within study centres adjusted for using cluster-robust variance estimators

**Supplemental Figure 1.** Glomerular hyperfiltration cut-offs according to age

 Abbreviation: eGFR = estimated glomerular filtration rate

**Supplemental Figure 2.** Study flowchart of patient selection

Abbreviation: GFR **=** glomerular filtration rate

**Supplemental Figure 3.** eGFR distributions according to glomerular filtration category

Abbreviation: eGFR = estimated glomerular filtration rate

**Supplemental Figure 4.** Subgroup analysis of the risk of vascular death according to hyperfiltration, age group, sex, atrial fibrillation and heart failure
